# Preservation of Renal Blood Flow by the Antioxidant EUK-134 in LPS-Treated Pigs

**DOI:** 10.3390/ijms16046801

**Published:** 2015-03-25

**Authors:** Sheldon Magder, Dimitrios G. Parthenis, Imad Al Ghouleh

**Affiliations:** 1 Critical Care Division, Royal Victoria Hospital, 687 Pine Ave W, Montreal, QC H3A 1A1, Canada; 2 Vascular Surgery of Nikaia, Peiraeous 18454, Greece; E-Mail: dparthenis@yahoo.gr; 3 Vascular Medicine Institute and Department of Pharmacology and Chemical Biology, University of Pittsburgh, Room E1228-2B, 200 Lothrop St, Pittsburgh, PA 15261, USA

**Keywords:** endotoxin, sepsis, superoxide dismutase, catalase, peroxynitrite, EUK-134

## Abstract

Sepsis is associated with an increase in reactive oxygen species (ROS), however, the precise role of ROS in the septic process remains unknown. We hypothesized that treatment with EUK-134 (manganese-3-methoxy *N*,*N*'-bis(salicyclidene)ethylene-diamine chloride), a compound with superoxide dismutase and catalase activity, attenuates the vascular manifestations of sepsis *in vivo*. Pigs were instrumented to measure cardiac output and blood flow in renal, superior mesenteric and femoral arteries, and portal vein. Animals were treated with saline (control), lipopolysaccharide (LPS; 10 µg·kg^−1^·h^−1^), EUK-134, or EUK-134 plus LPS. Results show that an LPS-induced increase in pulmonary artery pressure (PAP) as well as a trend towards lower blood pressure (BP) were both attenuated by EUK-134. Renal blood flow decreased with LPS whereas superior mesenteric, portal and femoral flows did not change. Importantly, EUK-134 decreased the LPS-induced fall in renal blood flow and this was associated with a corresponding decrease in LPS-induced protein nitrotyrosinylation in the kidney. PO_2_, pH, base excess and systemic vascular resistance fell with LPS and were unaltered by EUK-134. EUK-134 also had no effect on LPS-associated increase in CO. Interestingly, EUK-134 alone resulted in higher CO, BP, PAP, mean circulatory filling pressure, and portal flow than controls. Taken together, these data support a protective role for EUK-134 in the renal circulation in sepsis.

## 1. Introduction

Sepsis is a diffuse inflammatory response to an invading pathogen frequently associated with multi-organ injury and dysfunction [1,2], and characterized by inflammatory cell tissue infiltration, microthrombi and vascular leakage [3,4]. To date, there are no approved drugs to target sepsis and treatments are limited to nonspecific administration of intravenous fluids, antibiotics and oxygen in efforts to maintain organ function [1,5]. Of note is a marked increase in reactive oxygen species (ROS) and oxidative stress, which are hypothesized to play a role in sepsis [1,3,6]. However, studies investigating a causal link between oxidative-injury and organ malfunction had inconsistent findings. For example, pretreatment with superoxide dismutase (SOD) improved survival in rodents with endotoxemia [7,8,9] and protected against cardiac dysfunction [10], but failed to reduce respiratory dysfunction in sheep, pigs, dogs, or rabbits [11,12,13,14]. Oxygen radical scavengers, given alone or as adjuvant therapies, did however reduce lung injury [12,15,16,17,18,19,20,21] and improved hemodynamics and survival in some but not all studies [19,22,23,24,25].

Septic patients who were able to achieve normal plasma antioxidant potential had better survival [26], and treatment of septic patients with antioxidants glutathione and/or *N-*acetylcysteine (NAC) decreased oxidative injury [27,28], improved systemic oxygenation [29], and improved hepatic blood flow [30]. Nonetheless, NAC did not improve outcome in patients with acute lung injury [31,32]. Supplementation of feeds with antioxidant vitamins C and E showed some positive outcomes, resulting in less 28-day mortality in critically ill patients, reduced organ failure and shortened hospital stay [4,33]. More recently, levosimendan was suggested to exhibit protective antioxidant potential in a randomized trial [34]. While these studies are promising, clinical trials remain inconclusive [35].

Therapeutic trials of antioxidants may have failed because of inadequate subcellular targeting [35], and/or because the agents fail to alter cellular redox state. For example, an important limitation of NAC is that it works by increasing intracellular cysteine, which is already at very high levels relative to plasma. Thus, potentially toxic plasma levels are needed to raise intracellular levels. NAC also has a low Michaelis constant (*K*m) for removal of the major ROS, superoxide (O_2_^•−^). An interesting potential solution was demonstrated by Ortolani *et al.* who combined NAC and glutathione therapy in septic patients and showed a non-statistically significant trend towards less organ damage [28].

SOD and catalase are naturally occurring antioxidants, but these large molecules do not cross cell membranes and thus, given exogenously, do not affect intracellular ROS sources. While polyethylene glycol can facilitate cell entry of SOD, data remains inconclusive on the protective effects of this strategy in sepsis [10,12]. Furthermore, SOD alone, in the absence of catalase, may in theory act as a pro-oxidant by catalyzing the conversion of O_2_^•−^ to hydrogen peroxide (H_2_O_2_), which in turn is converted to hydroxyl radical, both potent ROS. To counter these problems, small molecules possessing both SOD and catalase activities were developed, including manganese-salen (Mn-salen) complexes EUK-8 (manganese *N*,*N*'-bis(salicyclidene)ethylene-diamine chloride), EUK-134 (manganese-3-methoxy *N*,*N*'-bis(salicyclidene)ethylene-diamine chloride), EUK-189 and EUK-207, that remove both O_2_^•−^ and H_2_O_2_ [36,37,38]. In rodent models, these reduced organ injury in endotoxic shock, hemorrhagic organ injury, renal ischemia-reperfusion injury, skeletal muscle atrophy, toxic-induced neuronal death, radiation injury and acute lung injury [36,37,38,39,40,41,42]. In porcine models, EUK compounds conferred hypoxic ischemia neuroprotection and attenuated acute lung injury in a model of adult respiratory distress syndrome [19,43]. Nonetheless, whether these compounds have potential to confer hemodynamic protection in large animal models of sepsis is a question that remains unanswered, significantly hindering evaluation of their translational relevance. Interestingly, we previously demonstrated *in vivo* that O_2_^•−^ production is increased in the vessels of pigs with endotoxin-induced shock [44] and established a role for increased O_2_^•−^ production in endotoxin-induced endothelial activation in *in vitro* models of sepsis [45,46]. Thus, in the current study we examine the potential hemodynamic benefits of EUK-134 in a porcine model of sepsis.

## 2. Results and Discussion

The major observation in this study is that EUK-134 blocked the LPS induced fall in renal blood flow and prevented the rise in pulmonary artery pressure. EUK-134 did not alter the fall in pH, base excess, and PO_2_ or the fall in systemic vascular resistance (SVR). All animals survived to the end of the planned four-hour study period except for one animal in the LPS group that died 220 min after LPS infusion.

### 2.1. The Effect of Lipopolysaccharide (LPS) on Blood Gases

**Figure 1:**
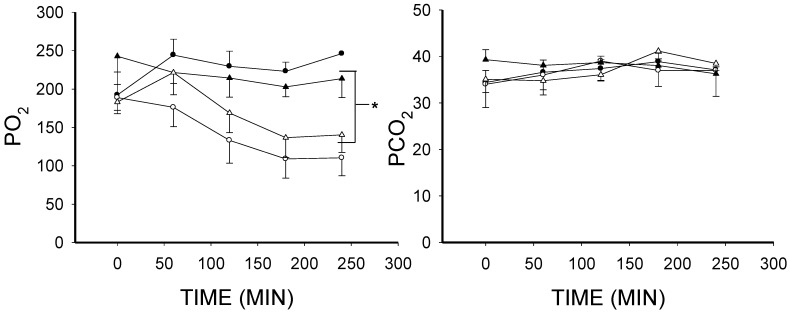

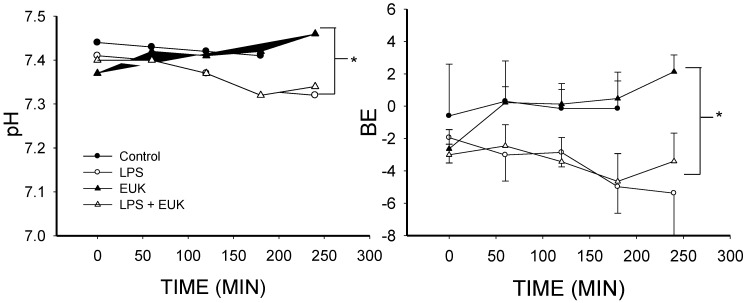
Mean arterial blood gases over time for the four conditions (mean ± SEM). **Upper left**, PO_2_ fell in the LPS and LPS plus EUK-134 (LPS + EUK) groups and did not change in the control or EUK-134 alone (EUK) groups; **Lower left**, pH fell in the LPS and LPS + EUK groups but did not change in the control or EUK groups; **Upper right**, PCO_2_ was comparable across all the groups (by design it was controlled by adjusting the ventilator); and **Lower right**, base excess (BE) fell in both the LPS and LPS + EUK groups, but not in control or EUK groups. * *p* < 0.001.

LPS treatment produced progressive falls in PO_2_, pH and base excess (BE; *p* < 0.001) and these were not prevented by EUK-134 treatment (Figure 1). PCO_2_ in all animals of all groups was kept constant by adjusting the ventilator (Figure 1), thereby controlling ventilatory function. Thus, the acidosis indicated by the drop in pH was purely metabolic. The fall in pH and BE relative to the controls also clearly indicates that there was a septic state [47,48,49,50].

### 2.2. EUK-134 Effect on LPS-Induced Hemodynamic Changes

**Figure 2:**
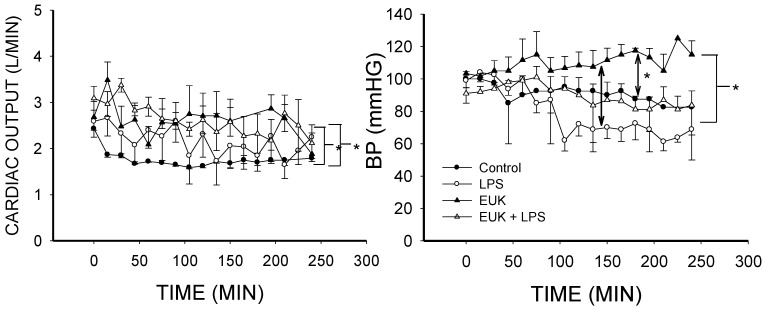
Cardiac output and blood pressure over time for the four conditions (mean ± SEM). **Left**, cardiac output (L/min) was higher in the LPS group and LPS plus EUK-134 groups than the control group (*p* < 0.02 and *p* < 0.005, respectively). Cardiac output in EUK-134 alone was also higher than control (*p* < 0.001) and LPS alone (*p* < 0.005); **Right**, blood pressure (BP) was lowest in the LPS group but was not significantly different from control (*p* < 0.068); This drop was rescued in the LPS plus EUK-134 group, which was superimposable with the control group. BP was best maintained or slightly rose in the EUK-134 group; This group was significantly greater than the LPS (*p* < 0.001), control (*p* < 0.001), and LPS plus EUK-134 group (*p* < 0.001). * *p* < 0.02, 0.005 or 0.001.

Figure 2 shows that cardiac output was higher in LPS-treated animals than controls (*p* < 0.02), in agreement with our previous studies [44,51]. Cardiac output was also higher in the LPS plus EUK-134-treated animals than in the control group (*p* < 0.005), and cardiac output in EUK-134 alone was greater than LPS-treated animals (*p* < 0.005) as well as the controls (*p* < 0.001). Cardiac output in this model is related to the volume given in that a minimum volume is required for preload preservation [52]. The volume resuscitation was based on the central venous pressure (CVP) measurement and the group means were similar with no statistical differences between groups (data not shown, see Section 3.4.). We did not adequately quantify the volume given in this study unfortunately. Nonetheless, these findings indicate that EUK-134 did not reverse the rise in cardiac output associated with LPS treatment.

Blood pressure was lowest in the LPS group, but despite a clear trend, the decrease in this group compared to the control group did not reach statistical significance (Figure 2), possibly due to volume resuscitation and variability among animals. Blood pressure in the LPS plus EUK-134 group was super-imposable with the control (Figure 2). Blood pressure was higher in the EUK-134 alone group than in the LPS and LPS plus EUK-134 animals (Figure 2; *p* < 0.001 in both cases). These data are in line with previous reports on EUK-8 in pigs [19] and rats [36], but differ from other studies in rodents [37,53]. Nonetheless, our findings suggest that EUK-134 may normalize the trend towards lower blood pressure following LPS treatment, but at the dose used, it seemed to have a small independent effect on blood pressure in non-diseased animals.

**Figure 3:**
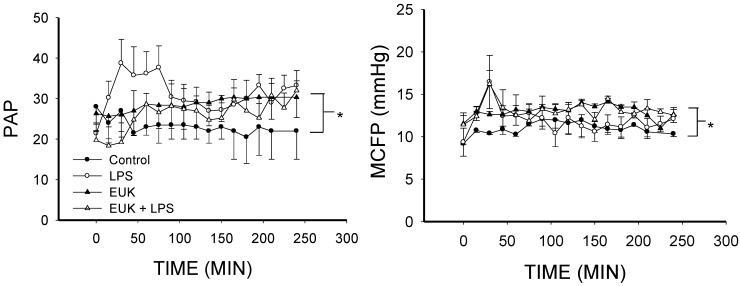
Pulmonary artery pressure (PAP) and mean circulatory filling pressure (MCFP) over time for the four conditions (mean ± SEM). **Left**, PAP in the LPS group was higher than control (*p* < 0.001). LPS plus EUK-134 (*p* < 0.001) and EUK alone (*p* < 0.02) were higher than the control but were not significantly different from each other. LPS plus EUK-134 was lower than LPS alone; **Right**, MCFP in LPS was not different from control although there appeared to be a “spike” in the pressure that was also seen with LPS plus EUK-134 and had the same timing as the rise in PAP. MCFP was greater in the EUK-134 alone group than in the control (*p* < 0.003) and LPS plus EUK-134 (*p* < 0.003). MCFP of the LPS plus EUK-134 group was greater than that of LPS (*p* < 0.014). * *p* < 0.02, 0.005 or 0.001.

Although we could not analyze the interaction, as in our previous studies, there was an initial rise in pulmonary artery pressure (PAP) in LPS animals, and the PAP in this group was significantly higher than control (*p* < 0.001) and LPS plus EUK-134 (*p* < 0.001) groups (Figure 3), supporting a rescue of the initial rise in PAP by EUK-134 in septic animals. Interestingly, PAP in the EUK alone group was also slightly higher than in the controls (Figure 3; *p* < 0.02), but this increase was lower than that in the LPS group, especially during the first 100 min.

The mean circulatory filling pressure (MCFP) in LPS treated animals was not different from controls (Figure 3). EUK-134 alone significantly increased MCFP (*p* < 0.003) compared to control as well as LPS treated animals (*p* < 0.001), and MCFP of the EUK-134 plus LPS group was greater than LPS alone (*p* < 0.01) as well as control (*p* < 0.001) as can be seen in Figure 3. Thus, despite similar CVP values, or even CVP values that tended to be on the lower side (data not shown), both EUK-134 alone and EUK-134 plus LPS treated animals had higher values of MCFP.

**Figure 4:**
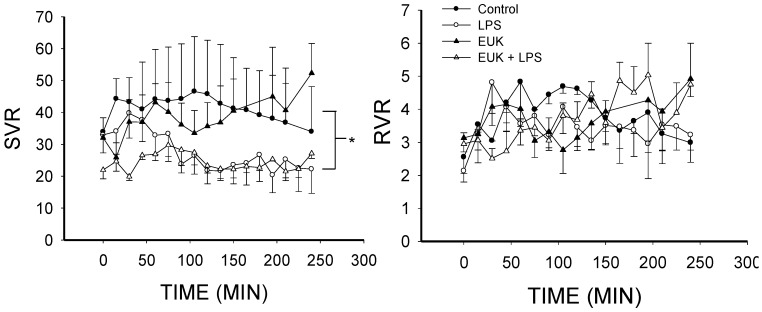
Systemic vascular resistance (SVR) and resistance to venous return (RVR) over time for the four conditions (mean ± SEM). **Left**, SVR fell in both LPS and LPS plus EUK-134 groups (*p* < 0.001). There were no differences between LPS and LPS plus EUK-134 nor between Control and EUK alone; **Right**, RVR was not different among the four groups. * *p* < 0.001.

Systemic vascular resistance (SVR) was decreased in both LPS- and LPS plus EUK-134-treated animals compared to control (*p* < 0.001), but SVR in the LPS plus EUK-134 group was maintained at more constant levels than the LPS alone group (Figure 4). Given that SVR is a function of blood pressure and cardiac output, this difference is likely related to better preservation of blood pressure in the LPS plus EUK-134 group since the cardiac output was similar in both group (Figure 2). SVR in the EUK alone animals was the same as in the controls (Figure 4), but occurred with a higher cardiac output and blood pressure (Figure 2). Resistance to venous return (RVR) did not differ among groups (Figure 4).

### 2.3. EUK-134 Attenuation of LPS-Induced Vascular Flow

**Figure 5:**
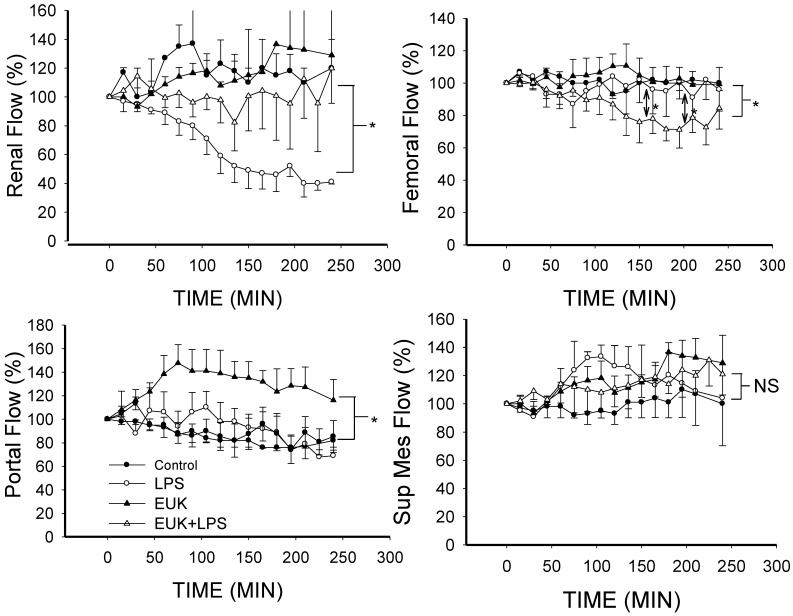
Mean blood flows in different vascular beds as a percent of baseline over time for the four conditions (mean ± SEM). **Upper left**, renal arterial flow; Renal artery flow was lower in the LPS group than the other three groups (*p* < 0.001 for each condition); **Lower left**, portal venous flow; Portal flow was higher in the EUK-134 alone group than in the other three conditions (*p* < 0.001); **Upper right**, femoral arterial flow; Femoral flow in the LPS plus EUK-134 group was lower than the other three groups (*p* < 0.001); and **Lower right**, superior mesenteric flow; There was no difference among the four conditions. * *p* < 0.001; NS: not statistically significant.

Our most striking observation was the reversal of LPS-induced decrease in renal blood flow (Figure 5). LPS produced a progressive sharp fall in renal artery blood flow compared to control (*p* < 0.001) and this was entirely prevented by EUK-134 (*p* < 0.001 for LPS group *vs.* LPS plus EUK). There was no difference in renal flow between the EUK alone group and control (Figure 5). This protective effect appeared to be unique to the renal circulation as blood flow in the femoral artery did not change with LPS alone nor EUK-134 alone. Interestingly, femoral flow fell when LPS treated pigs were also given EUK-134 (Figure 5). This may be explained by effects of EUK-134 on central-to-peripheral vascular tone decoupling in sepsis [54]. Under septic conditions, central and peripheral vascular impedance and compliance change in a manner that even though flow may be maintained, central *vs.* peripheral pressure differences are perturbed. Treatment with EUK-134 may restore these differences by affecting vascular tone to different extents in central and peripheral vessels such as the femoral artery. This may result in restoration of peripheral to central shunting and thereby a reduction in peripheral flow.

There was a tendency for flow to decrease in the portal vein over time with all conditions except EUK-134 alone in which case the flow was significantly greater than in the other conditions (Figure 5). Furthermore, there were no significant changes in flow in the superior mesenteric artery in any of the conditions (Figure 5). These findings of a protective role in the kidney are supported by previous studies in rodent models that demonstrated that EUK-134 reduces the rise in creatinine and urea in response to LPS in rats [36,37]. Moreover, in an ischemia-reperfusion model in rats, EUK-134 reduced the rise in serum creatinine, urea and tubular damage, and maintained creatinine clearance and fractional excretion of Na^+^ [39].

**Figure 6:**
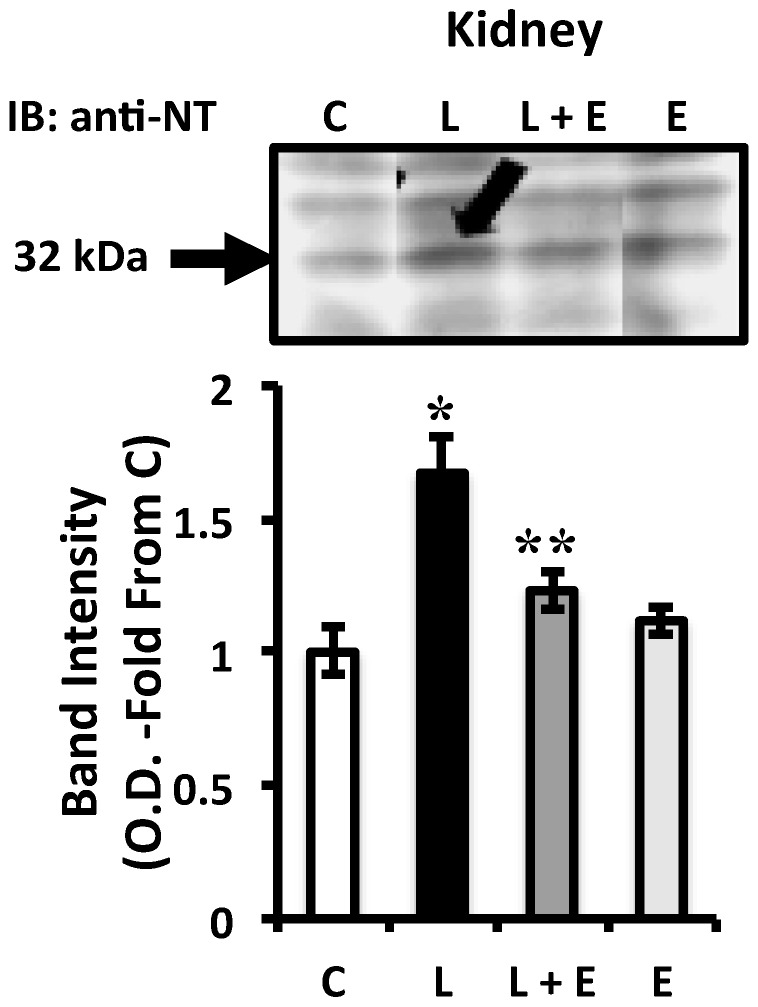
Western blots with anti-nitrotyrosine antibody of homogenates of renal tissue. There was an increase density at a band at 32 kDa (marked by arrow) with LPS and this was not apparent when animals were pretreated with EUK-134. Bar graph represents relative band optical density (OD) quantification. C = Control, L = LPS, L + E = LPS plus EUK-134 and E = EUK-134; NT = Nitrotyrosine. * *p* < 0.05 *vs.* C; ** *p* < 0.05 *vs.* L.

### 2.4. Attenuation of LPS-Induced Renal Oxidative Stress by EUK-134 Treatment

To assess whether the protective effect of EUK-134 on renal flow is a consequence of an attenuation of kidney tissue oxidative damage, we analyzed the level of nitrotyrosine formation by Western blot in renal tissues from the four animal groups. Nitrotyrosine is a foot-print of peroxynitrite, the product of nitric oxide and O_2_^•−^ and a potent oxidant that can be very toxic to tissues [55]. Figure 6 shows that nitrotyrosine staining was increased in LPS treated animals compared to control (*p* < 0.05), in agreement with our previous reports [51,56]. Importantly, this increase was prevented by EUK-134 treatment in the LPS plus EUK group (*p* < 0.05). These findings are consistent with studies evaluating EUK compounds [36,37] and peroxynitrite or O_2_^•−^ scavengers [57] in rat models, and with the notion that scavenging ROS in sepsis improves outcome by decreasing the production of peroxynitrite. Indeed, we have previously shown that peroxynitrite production is likely related to increased NO from endogenous nitric oxide synthase and increased O_2_^•−^ production [51,56] and that there is increased production of O_2_^•−^ in carotid arteries [44] and ventilatory muscles [58] of pigs treated with the same protocol as in this current study. Thus, although not measured, it is likely that O_2_^•−^ was also increased in this study. In our previous studies, we also showed that the increase in O_2_^•−^ occurred through activation of NADPH oxidase both in vessels and in ventilatory muscles [44,58]. Our *in vitro* studies on LPS-treated endothelial cells extended these findings to implicate the increase in NADPH oxidase-derived O_2_^•−^ in endothelial activation, a critical component of the systemic septic response [45,46]. It is likely, therefore, that the protective effects observed in renal tissues by EUK-134 are a result of scavenging of NADPH oxidase-derived O_2_^•−^.

### 2.5. General Discussion

Taken together, our findings demonstrate that EUK-134 attenuates the decrease in renal flow and rise in PAP in sepsis and suggest that it may also stabilize blood pressure and dampen variability in SVR over time. Our data also demonstrate that this occurs, at least in part, via a reduction in oxidative tissue injury resulting from peroxynitrite formation in the kidney. The novelty of our findings can be appreciated when considering the translational potential to human disease. Previous studies examining the role of manganese-salen compounds in endotoxin exposure were performed in rodents [36,37]. Rodent models are limited in that substantial differences exist between them and higher mammalian systems which may preclude translation to human disease. For example, inducible nitric oxide plays a much smaller role in non-rodents [51] and constitutive nitric oxide can play a greater role [59]. Indeed, the only other study that has examined the role of a manganese-salen compound in endotoxic shock in a non-rodent species was by Gonzalez *et al.* who examined the effect of EUK-8 on lung injury [19]. While Gonzalez *et al.* focused on modeling lung injury in acute adult respiratory distress syndrome by giving an initial moderate dose of LPS (20 μg·kg^−1^ IV bolus) followed 18 h later by a large dose (250 μg·kg^−1^ over an hour), and used the less potent EUK-8 [60], some comparisons to our study can be made. In their study as in ours and our previous studies [52], LPS produced a rise in pulmonary artery pressure which was especially marked in the first 50 min and this was attenuated by EUK-8 in their study and EUK-134 in ours. A major observation in their study was that EUK-8 attenuated the fall in PO_2_ with LPS but EUK-134 did not prevent the fall in PO_2_ in our study. Gonzalez *et al.* did not report other measures of arterial blood gases [19].

One limitation of our study was that we did not adequately quantify the volume of fluid resuscitation but used the value of CVP as a guide. While CVP may be criticized due mainly to measurement errors, in this study we were careful to reference CVP measurement from the midpoint of the right atrium, which is the most accepted point [61]. Moreover, CVP was consistently measured at end-expiration, the point at which pleural pressure is closest to atmospheric pressure, allowing for more accurate CVP measurement [61]. Maintenance of a constant CVP is sometimes used as an indicator of preload preservation and a parameter to gauge that volume given was similar across groups. This is typically meant to obtain an indirect confirmation that any changes observed in cardiac output are due to cardiac function and not the function governing blood return from the vascular reservoir [61]. Nonetheless, recent studies indicate that the use of CVP as a guide for fluid therapy should be avoided [62]. Thus, while our findings with respect to cardiac output are in line with our previous studies in this model [44,51,52,59], we cannot entirely rule out differences in volume resuscitation between groups to be a contributing factor to the observed measurements on cardiac output in this study.

EUK-134 did appear to have effects on its own. For example, treatment with EUK-134 alone affected CO, MCFP and portal flow. This may be related to the antioxidant capacity of EUK-134, since ROS are known to have homeostatic functions in addition to their roles in disease [63,64]. Nonetheless, treatment of EUK-134 alone had no effect on renal flow compared to control, but reversed the LPS-associated reduction. Thus, the protective effects of EUK-134 on LPS-associated reduction in renal flow, are in specific relation to LPS treatment and not a result of EUK alone having an effect.

Measurements of changes in blood flow to the splanchnic bed in sepsis have produced variable results that are likely dependent upon the model. In most animal models of sepsis, cardiac output is low and SVR high and in these models, splanchnic flow is decreased [65,66,67]. In contrast, splanchnic flow was increased in animal models in which there was a hyperdynamic circulatory response [68], or at least a decrease in SVR [69,70]. Splanchnic flow was also found to be elevated in septic patients relative to patients who had undergone cardiac surgery [71]. In our animals, with a small increase in cardiac output with LPS and a fall in SVR, there was no change in superior mesenteric or portal flow compared to controls or to baseline, consistent with previous studies in hyperdynamic animals. Somewhat surprising was the marked rise in portal flow with administration of EUK-134 alone. This suggests that this bed is relatively constricted at basal conditions by the presence of reactive oxygen species.

## 3. Experimental Section

### 3.1. General Procedure

All procedures were performed according to the guidelines of the animal care committee of McGill University. Domestic pigs (*n* = 15) weighing 29.0 ± 4.2 (range 25.4–32.8 kg) were sedated with ketamine 30 mg·kg^−1^, atropine 1.0 mg·kg^−1^ and xylazine 2 mg·kg^−1^. Twenty minutes later, they were anesthetized with 10–15 mg·kg^−1^ of sodium thiopental; Anesthesia was maintained with a continuous intravenous infusion of sodium thiopental at 5 mg·kg^−1^·h^−1^. The animals were placed in the supine position in a V-shaped support, intubated with a cuffed endotracheal tube, and ventilated with a volume respirator at a tidal volume of 12 mL·kg^−1^ and a frequency of 12–15 breaths·min^−1^ and 5 cm of water positive end-expiratory pressure. Through a midline (right lateral) incision in the neck, the left common carotid artery was isolated and cannulated with a polyethylene catheter for pressure measurements. The right internal jugular vein was isolated and a pulmonary artery flotation catheter was passed into the pulmonary artery. A 12F balloon-tipped catheter with a 50-cc capacity (Prewitt aortic occlusion catheter #10, Pramel Inc., Longueuil, QC, Canada) was placed in the right atrium through the external jugular vein. When inflated to 40 cc, this balloon transiently obstructed the circulation and was used to stop the venous return and measure the mean circulatory filling pressure (MCFP). The right femoral vein was cannulated with a polyethylene catheter (Cole Palmer, Anjou, QC, Canada) for the administration of drugs. Blood gases were monitored, PCO_2_ was kept between 30 and 40 mmHg by adjusting the ventilator, and PO_2_ at greater than 90 mmHg by giving supplemental oxygen. Cardiac output was measured by the thermodilution method (Abbott 3300, North Chicago, Chicago, IL, USA) by injecting 3 mL of 5% dextrose in water at room temperature into the right atrial port of the pulmonary catheter.

### 3.2. Measurement of MCFP

To measure mean circulatory filling pressure, the balloon in the right atrium was rapidly inflated with 40 cc of air for 15–20 s [52]. This transiently arrests venous return, and the venous pressure measured in the central vein becomes equal to the pressure upstream in the compliant region of the venous system. This procedure could be repeated frequently without an effect on the hemodynamic parameters or general condition of the animal. It was reproducible with <0.5 mmHg difference on repeated measurements under the same conditions. The measurement was obtained after 15 s because this avoids reflex changes, although the arterial plateau pressure (APP) remains above the venous plateau pressure (VPP). The APP remains high because volume continues to drain through the high arterial resistance and also because there is an arterial critical closing pressure, which traps volume in the arterial vessels [52]. This volume is accounted for by the formula:

*MCFP = VPP + (APP − VPP) ×* (arterial compliance/venous compliance)

where the ratio of arterial to venous compliance is assumed to be 1:30.

### 3.3. Flow-Measurement

Left renal, left femoral, superior mesenteric arteries and portal vein flows were measured by placing Doppler flow probes of appropriate size around the vessels [72]. The probes were connected to a multi-channel single-pulse Doppler flowmeter (Valpey-Fisher, Hopkinton, MA, USA). Zero baseline flows were determined by occlusion of the vessels proximal to the flow probes. In this study, the absolute flow was not assessed and flows are reported as a percentage of the baseline flow so as to determine the change over time.

### 3.4. Protocol

Pigs were given sufficient volume (normal saline) to maintain a central venous pressure (CVP) greater than 2 mmHg referenced to the mid-portion of the right atrium. There were four groups of animals: (1) A time control that received saline only (*n* = 2); (2) LPS treated pigs that received 10 μg·kg^−1^·h^−1^ of LPS for 2 h and then were followed for another 2 h (*n* = 5) as in our previous studies [44,51,59]; (3) Pigs given 0.5 mg·kg^−1^ of EUK-134 alone followed by a second bolus of 1.0 mg·kg^−1^ one hour later (*n* = 3); and (4) Pigs given the same dose of EUK-134 and followed 15 min later by the LPS infusion at 10 μg·kg^−1^·h^−1^ for 2 h and followed for another 2 h (*n* = 5). The order of treatments was randomized.

### 3.5. Hemodynamic Calculations

The systemic vascular resistance (*SVR*) was calculated as *SVR* = (*Part − CVP*)/*CO*, where *Part* is mean arterial blood pressure, *CVP* is central venous pressure and CO is cardiac output in l min^−1^. Pulmonary vascular resistance (*PVR*) was calculated from *PVR =* (*Ppa − Pcw*)*/CO*, where *Ppa* is mean pulmonary artery pressure and *Pcw* is the pulmonary capillary wedge pressure. Resistance to venous return (*RVR*) was calculated from *RVR =* (*MCFP − Pra*)*/CO*, where *Pra* is right atrial pressure. All these are in the units of mmHg·L^−1^ min.

### 3.6. Western Blot Analysis for Nitrotyrosine

Frozen tissue extracts (*n* = 2) were thawed on ice, homogenized and mixed with equal volume of sample buffer (composed of 4 mL distilled water, 1 mL of 0.5 M Tris-HCl, pH = 6.8, 0.8 mL glycerol, 10% SDS (*w*/*v*), 0.05% (*w*/*v*) bromophenol blue, 0.4 mL of 2-β-mercaptoethanol) followed by heating at 95 °C for 5 min. They were centrifuged and loaded on 8% or 4%–12% Tris/glycine/SDS-polyacrylamide gel for fractionation. Predetermined MW standards (Novex Inc., Wadswort, OH, USA) were used as markers. Protein on the gel was transferred onto PVDF membranes at 4 °C at 25–30 V and 370–380 mA for 150 min. After transfer, the membranes were incubated with 5% skim milk, in wash buffer for 2 h, or overnight, at 4 °C. Nitrotyrosine was detected with anti-nitrotyrosine monoclonal antibody (Cat # 189542, Cayman Chemical Co., Ann Arbor, MI, USA). The membranes were washed three times with wash buffer and then incubated with anti-mouse antibody (1:2000 or 10,000). Immuno-reactive bands were visualized by enhanced chemiluminescence (ECL kit, Pierce Biotechnology, Rockford, IL, USA according to the specifications of the manufacturer (Amersham, Chiltern, UK). Blots were scanned with an imaging densitometer, and the optical densities of protein bands were quantified with software (Sigma Gel, Jandel Scientific, San Rafael, CA, USA).

### 3.7. Statistics

Data are presented as mean ± standard error of the mean (SEM). Data was analyzed with a two-way analysis of variance for treatment condition and time. *Post hoc* analysis was performed with a Tukey test for multiple comparisons. *p* < 0.05 was considered significant.

## 4. Conclusions

In conclusion, pretreatment of pigs with EUK-134 prevented the fall in renal blood flow induced by LPS. This was associated with evidence of a decrease in nitrosative stress in the kidney supporting a renal protective effect. EUK-134 also blunted the rise in pulmonary artery pressure but did not prevent the fall in SVR nor the development of hypoxia and metabolic acidosis. These data demonstrate the promise of SOD/catalase mimetics as potential therapeutic agents for the targeting some of the vascular impairments of endotoxic shock. More work is needed to test these agents in combination with current practices to combat the symptoms of shock in the clinic.

## References

[1] Deutschman C.S., Tracey K.J. (2014). Sepsis: Current dogma and new perspectives. Immunity.

[2] Russell J.A. (2006). Management of sepsis. N. Engl. J. Med..

[3] Salvemini D., Cuzzocrea S. (2002). Oxidative stress in septic shock and disseminated intravascular coagulation. Free Radic. Biol. Med..

[4] Wilson J.X. (2013). Evaluation of vitamin C for adjuvant sepsis therapy. Antioxid. Redox Signal..

[5] De Backer D., Scolletta S. (2013). Clinical management of the cardiovascular failure in sepsis. Curr. Vasc. Pharmacol..

[6] Goode H.F., Webster N.R. (1993). Free radicals and antioxidants in sepsis. Crit. Care Med..

[7] Warner B.W., Hasselgren P.O., Fischer J.E. (1986). Effect of allopurinol and superoxide dismutase on survival rate in rats with sepsis. Curr. Surg..

[8] Kunimoto F., Morita T., Ogawa R., Fujita T. (1987). Inhibition of lipid peroxidation improves survival rate of endotoxemic rats. Circ. Shock.

[9] Schneider J., Friderichs E., Giertz H. (1989). Protection by recombinant human superoxide dismutase in lethal rat endotoxemia. Prog. Clin. Biol. Res..

[10] Supinski G.S., Callahan L.A. (2006). Polyethylene glycol-superoxide dismutase prevents endotoxin-induced cardiac dysfunction. Am. J. Respir. Crit. Care Med..

[11] Traber D.L., Adams T., Sziebert L., Stein M., Traber L. (1985). Potentiation of lung vascular response to endotoxin by superoxide dismutase. J. Appl. Physiol..

[12] Olson N.C., Grizzle M.K., Anderson D.L. (1987). Effect of polyethylene glycol-superoxide dismutase and catalase on endotoxemia in pigs. J. Appl. Physiol..

[13] Novotny M.J., Laughlin M.H., Adams H.R. (1988). Evidence for lack of importance of oxygen free radicals in *Escherichia coli* endotoxemia in dogs. Am. J. Physiol..

[14] Broner C.W., Shenep J.L., Stidham G.L., Stokes D.C., Fairclough D., Schonbaum G.R., Rehg J.E., Hildner W.K. (1989). Effect of antioxidants in experimental *Escherichia coli* septicemia. Circ. Shock.

[15] Olson N.C., Anderson D.L., Grizzle M.K. (1987). Dimethylthiourea attenuates endotoxin-induced acute respiratory failure in pigs. J. Appl. Physiol..

[16] Seekamp A., Lalonde C., Zhu D.G., Demling R. (1988). Catalase prevents prostanoid release and lung lipid peroxidation after endotoxemia in sheep. J. Appl. Physiol..

[17] Petrone W.F., English D.K., Wong K., McCord J.M. (1980). Free radicals and inflammation: Superoxide-dependent activation of a neutrophil chemotactic factor in plasma. Proc. Natl. Acad. Sci. USA.

[18] Bernard G.R., Lucht W.D., Niedermeyer M.E., Snapper J.R., Ogletree M.L., Brigham K.L. (1984). Effect of *N*-acetylcysteine on the pulmonary response to endotoxin in the awake sheep and upon *in vitro* granulocyte function. J. Clin. Investig..

[19] Gonzalez P.K., Zhuang J., Doctrow S.R., Malfroy B., Benson P.F., Menconi M.J., Fink M.P. (1995). EUK-8, a synthetic superoxide dismutase and catalase mimetic, ameliorates acute lung injury in endotoxemic swine. J. Pharmacol. Exp. Ther..

[20] Galvao A.M., Wanderley M.S., Silva R.A., Filho C.A., Melo-Junior M.R., Silva L.A., Streck E.L., Dornelas de Andrade A.F., Souza Maia M.B., Barbosa de Castro C.M. (2014). Intratracheal co-administration of antioxidants and ceftriaxone reduces pulmonary injury and mortality rate in an experimental model of sepsis. Respirology.

[21] Campos R., Shimizu M.H., Volpini R.A., de Braganca A.C., Andrade L., Lopes F.D., Olivo C., Canale D., Seguro A.C. (2012). *N*-acetylcysteine prevents pulmonary edema and acute kidney injury in rats with sepsis submitted to mechanical ventilation. Am. J. Physiol. Lung Cell. Mol. Physiol..

[22] Saetre T., Hoiby E.A., Aspelin T., Lermark G., Egeland T., Lyberg T. (2000). Aminoethyl-isothiourea, a nitric oxide synthase inhibitor and oxygen radical scavenger, improves survival and counteracts hemodynamic deterioration in a porcine model of streptococcal shock. Crit. Care Med..

[23] Brandes R.P., Koddenberg G., Gwinner W., Kim D., Kruse H.J., Busse R., Mugge A. (1999). Role of increased production of superoxide anions by NAD(P)H oxidase and xanthine oxidase in prolonged endotoxemia. Hypertension.

[24] Tsao C.M., Jhang J.G., Chen S.J., Ka S.M., Wu T.C., Liaw W.J., Huang H.C., Wu C.C. (2014). Adjuvant potential of selegiline in attenuating organ dysfunction in septic rats with peritonitis. PLoS ONE.

[25] Maurya H., Mangal V., Gandhi S., Prabhu K., Ponnudurai K. (2014). Prophylactic antioxidant potential of gallic acid in murine model of sepsis. Int. J. Inflamm..

[26] Cowley H.C., Bacon P.J., Goode H.F., Webster N.R., Jones J.G., Menon D.K. (1996). Plasma antioxidant potential in severe sepsis: A comparison of survivors and nonsurvivors. Crit. Care Med..

[27] Heller A.R., Groth G., Heller S.C., Breitkreutz R., Nebe T., Quintel M., Koch T. (2001). *N*-acetylcysteine reduces respiratory burst but augments neutrophil phagocytosis in intensive care unit patients. Crit. Care Med..

[28] Ortolani O., Conti A., de Gaudio A.R., Moraldi E., Cantini Q., Novelli G. (2000). The effect of glutathione and *N*-acetylcysteine on lipoperoxidative damage in patients with early septic shock. Am. J. Respir. Crit. Care Med..

[29] Suter P.M., Domenighetti G., Schaller M.D., Laverriere M.C., Ritz R., Perret C. (1994). *N*-acetylcysteine enhances recovery from acute lung injury in man. A randomized, double-blind, placebo-controlled clinical study. Chest.

[30] Rank N., Michel C., Haertel C., Lenhart A., Welte M., Meier-Hellmann A., Spies C. (2000). *N*-acetylcysteine increases liver blood flow and improves liver function in septic shock patients: Results of a prospective, randomized, double-blind study. Crit. Care Med..

[31] Bernard G.R., Wheeler A.P., Arons M.M., Morris P.E., Paz H.L., Russell J.A., Wright P.E. (1997). A trial of antioxidants *N*-acetylcysteine and procysteine in ARDS. The Antioxidant in ARDS Study Group. Chest.

[32] Jepsen S., Herlevsen P., Knudsen P., Bud M.I., Klausen N.O. (1992). Antioxidant treatment with *N*-acetylcysteine during adult respiratory distress syndrome: A prospective, randomized, placebo-controlled study. Crit. Care Med..

[33] Crimi E., Liguori A., Condorelli M., Cioffi M., Astuto M., Bontempo P., Pignalosa O., Vietri M.T., Molinari A.M., Sica V. (2004). The beneficial effects of antioxidant supplementation in enteral feeding in critically ill patients: A prospective, randomized, double-blind, placebo-controlled trial. Anesth. Analg..

[34] Torraco A., Carrozzo R., Piemonte F., Pastore A., Tozzi G., Verrigni D., Assenza M., Orecchioni A., D’Egidio A., Marraffa E. (2014). Effects of levosimendan on mitochondrial function in patients with septic shock: A randomized trial. Biochimie.

[35] Galley H.F. (2010). Bench-to-bedside review: Targeting antioxidants to mitochondria in sepsis. Crit. Care.

[36] McDonald M.C., di Villa Bianca R.E., Wayman N.S., Pinto A., Sharpe M.A., Cuzzocrea S., Chatterjee P.K., Thiemermann C. (2003). A superoxide dismutase mimetic with catalase activity (EUK-8) reduces the organ injury in endotoxic shock. Eur. J. Pharmacol..

[37] Bianca R., Wayman N.S., McDonald M.C., Pinto A., Shape M.A., Chatterjee P.K., Thiemermann C. (2002). Superoxide dismutase mimetic with catalase activity, EUK-134, attenuates the multiple organ injury and dysfunction caused by endotoxin in the rat. Med. Sci. Monit..

[38] Rosenthal R.A., Fish B., Hill R.P., Huffman K.D., Lazarova Z., Mahmood J., Medhora M., Molthen R., Moulder J.E., Sonis S.T. (2011). Salen Mn complexes mitigate radiation injury in normal tissues. Anticancer Agents Med. Chem..

[39] Chatterjee P.K., Patel N.S., Kvale E.O., Brown P.A., Stewart K.N., Mota-Filipe H., Sharpe M.A., di Paola R., Cuzzocrea S., Thiemermann C. (2004). EUK-134 reduces renal dysfunction and injury caused by oxidative and nitrosative stress of the kidney. Am. J. Nephrol..

[40] Gianello P., Saliez A., Bufkens X., Pettinger R., Misseleyn D., Hori S., Malfroy B. (1996). EUK-134, a synthetic superoxide dismutase and catalase mimetic, protects rat kidneys from ischemia-reperfusion-induced damage. Transplantation.

[41] Rong Y., Doctrow S.R., Tocco G., Baudry M. (1999). EUK-134, a synthetic superoxide dismutase and catalase mimetic, prevents oxidative stress and attenuates kainate-induced neuropathology. Proc. Natl. Acad. Sci. USA.

[42] Lawler J.M., Kunst M., Hord J.M., Lee Y., Joshi K., Botchlett R.E., Ramirez A., Martinez D.A. (2014). EUK-134 ameliorates nNOSμ translocation and skeletal muscle fiber atrophy during short-term mechanical unloading. Am. J. Physiol. Regul. Integr. Comp. Physiol..

[43] Ni X., Yang Z.J., Carter E.L., Martin L.J., Koehler R.C. (2011). Striatal neuroprotection from neonatal hypoxia-ischemia in piglets by antioxidant treatment with EUK-134 or edaravone. Dev. Neurosci..

[44] Javeshghani D., Hussain S.N.A., Scheidel J., Quinn M.T., Magder S.A. (2003). Superoxide production in the vasculature of lipopolysaccharide treated rats and pigs. Shock.

[45] Al Ghouleh I., Magder S. (2008). Nicotinamide adenine dinucleotide phosphate (reduced form) oxidase is important for LPS-induced endothelial cell activation. Shock.

[46] Al Ghouleh I., Magder S. (2012). NADPH oxidase-derived superoxide destabilizes lipopolysaccharide-induced interleukin 8 mRNA via p38, extracellular signal-regulated kinase mitogen-activated protein kinase, and the destabilizing factor tristetraprolin. Shock.

[47] Gattinoni L., Carlesso E. (2013). Supporting hemodynamics: What should we target? What treatments should we use?. Crit. Care.

[48] Garcia X., Pinsky M.R. (2011). Clinical applicability of functional hemodynamic monitoring. Ann. Intensive Care.

[49] Kellum J.A. (2007). Disorders of acid-base balance. Crit. Care Med..

[50] Magder S. (2009). Bench-to-bedside review: Ventilatory abnormalities in sepsis. Crit. Care.

[51] Javeshghani D., Magder S. (2001). Presence of nitrotyrosine with minimal iNOS induction in LPS treated pigs. Shock.

[52] Magder S., Vanelli G. (1996). Circuit factors in the high cardiac output of sepsis. J. Crit. Care.

[53] Izumi M., McDonald M.C., Sharpe M.A., Chatterjee P.K., Thiemermann C. (2002). Superoxide dismutase mimetics with catalase activity reduce the organ injury in hemorrhagic shock. Shock.

[54] Hatib F., Jansen J.R., Pinsky M.R. (2011). Peripheral vascular decoupling in porcine endotoxic shock. J. Appl. Physiol..

[55] Szabo C., Saunders C., O’Connor M., Salzman A.L. (1997). Peroxynitrite causes energy depletion and increases permeability via activation of poly (ADP-ribose) synthetase in pulmonary epithelial cells. Am. J. Respir. Cell Mol. Biol..

[56] Mehta S., Javeshghani D., Datta P., Levy R.D., Magder S. (1999). Porcine endotoxaemic shock is associated with increased expired nitric oxide. Crit. Care Med..

[57] Salvemini D., Riley D.P., Lennon P.J., Wang Z.Q., Currie M.G., Macarthur H., Misko T.P. (1999). Protective effects of a superoxide dismutase mimetic and peroxynitrite decomposition catalysts in endotoxin-induced intestinal damage. Br. J. Pharmacol..

[58] Javesghani D., Magder S.A., Barreiro E., Quinn M.T., Hussain S.N. (2002). Molecular characterization of a superoxide-generating NAD(P)H oxidase in the ventilatory muscles. Am. J. Respir. Crit. Care Med..

[59] Javeshghani D., Magder S. (2001). Regional changes in constitutive nitric oxide synthase and the hemodynamic consequences of its inhibition in lipopolysaccharide-treated pigs. Shock.

[60] Doctrow S.R., Huffman K., Marcus C.B., Tocco G., Malfroy E., Adinolfi C.A., Kruk H., Baker K., Lazarowych N., Mascarenhas J. (2002). Salen-manganese complexes as catalytic scavengers of hydrogen peroxide and cytoprotective agents: Structure-activity relationship studies. J. Med. Chem..

[61] Magder S. (2006). Central venous pressure: A useful but not so simple measurement. Crit. Care Med..

[62] Marik P.E., Baram M., Vahid B. (2008). Does central venous pressure predict fluid responsiveness? A systematic review of the literature and the tale of seven mares. Chest.

[63] Al Ghouleh I., Khoo N.K., Knaus U.G., Griendling K.K., Touyz R.M., Thannickal V.J., Barchowsky A., Nauseef W.M., Kelley E.E., Bauer P.M. (2011). Oxidases and peroxidases in cardiovascular and lung disease: New concepts in reactive oxygen species signaling. Free Radic. Biol. Med..

[64] Bedard K., Krause K.H. (2007). The NOX family of ROS-generating NADPH oxidases: Physiology and pathophysiology. Physiol. Rev..

[65] Bressack M.A., Morton N.S., Hortop J. (1987). Group B streptococcal sepsis in the piglet: Effects of fluid therapy on venous return, organ edema, and organ blood flow. Circ. Res..

[66] Hussain S.N., Roussos C. (1985). Distribution of respiratory muscle and organ blood flow during endotoxic shock in dogs. J. Appl. Physiol..

[67] Van Lambalgen A.A., Bronsveld W., van den Bos G.C., Thijs L.G. (1984). Distribution of cardiac output, oxygen consumption and lactate production in canine endotoxin shock. Cardiovasc Res..

[68] Lang C.H., Bagby G.J., Ferguson J.L., Spitzer J.J. (1984). Cardiac output and redistribution of organ blood flow in hypermetabolic sepsis. Am. J. Physiol..

[69] Breslow M.J., Miller C.F., Parker S.D., Walman A.T., Traystman R.J. (1987). Effect of vasopressors on organ blood flow during endotoxin shock in pigs. Am. J. Physiol..

[70] Wyler F., Forsyth R.P., Nies A.S., Neutze J.M., Melmon K.L. (1969). Endotoxin-induced regional circulatory changes in the unanesthetized monkey. Circ. Res..

[71] Ruokonen E., Takala J., Kari A., Saxen H., Mertsola J., Hansen E.J. (1993). Regional blood flow and oxygen transport in septic shock. Crit. Care Med..

[72] Hussain S.N., Chatillon A., Comtois A., Roussos C., Magder S. (1991). Chemical activation of thin-fiber phrenic afferents. 2. Cardiovascular responses. J. Appl. Physiol..

